# Genomic diversity dynamics in conserved chicken populations are revealed by genome-wide SNPs

**DOI:** 10.1186/s12864-018-4973-6

**Published:** 2018-08-09

**Authors:** Mengmeng Zhang, Wei Han, Hui Tang, Guohui Li, Minjie Zhang, Ran Xu, Yijun Liu, Tao Yang, Wenting Li, Jianmin Zou, Keliang Wu

**Affiliations:** 1 Department of Animal Genetics and Breeding, National Engineering Laboratory for Animal Breeding, College of Animal Science and Technology, China Agricultural University, Beijing, 100193 People’s Republic of China; 2 National Chickens Genetic Resources, Institute of Poultry Science, Chinese Academy of Agricultural Science, Yangzhou, 225125 People’s Republic of China; 3 College of Animal Science and Technology, Shandong Agricultural University, Tai’an, 271018 People’s Republic of China; 4 College of Animal Sciences and Veterinary Medicine, Henan Agricultural University, Zhengzhou, 450002 People’s Republic of China; 5 Beijing Key Laboratory for Animal Genetic Improvement, Beijing, 100193 People’s Republic of China; 6 College of Animal Science, Southwest University, Chongqing, 402460 People’s Republic of China

**Keywords:** Chinese indigenous chicken, Genomic diversity, Conservation scheme, Genome-wide SNPs

## Abstract

**Background:**

Maintaining maximum genetic diversity and preserving breed viability in conserved populations necessitates the rigorous evaluation of conservation schemes. Three chicken breeds (Baier Yellow Chicken (BEC), Beijing You Chicken (BYC) and Langshan Chicken (LSC)) are currently in conservation programs in China. Changes in genetic diversity were measured by heterozygosity, genomic inbreeding coefficients, and autozygosity, using estimates derived from runs of homozygosity (ROH) that were identified using SNPs.

**Results:**

Ninety DNA samples were collected from three generations for each breed. In the most recent generation, the highest genetic diversity was observed in LSC, followed by BEC and BYC. Inbreeding coefficients based on ROH for the three breeds declined slightly between the first and middle generations, and then rapidly increased. No inbreeding coefficients exceeded 0.1. Population structure assessments using neighbor-joining tree analysis, principal components analysis, and STRUCTURE analysis indicated that no genetic differentiation existed within breeds. LD decay and ROH at different cut-off lengths were used to identify traces left by recent or ancient inbreeding. Few long ROH were identified, indicating that inbreeding has been largely avoided with the current conservation strategy. The observed losses in genetic diversity and occurrences of inbreeding might be consequences of sub-optimal effective population sizes.

**Conclusions:**

The conserved Chinese chicken populations have high genomic diversity under the current conservation program (R: F). Furthermore, this study highlights the need to monitor dynamic changes in genetic diversity in conserved breeds over successive generations. Our research provides new insights into genetic diversity dynamics in conserved populations, and lays a solid foundation for improving conservation schemes.

**Electronic supplementary material:**

The online version of this article (10.1186/s12864-018-4973-6) contains supplementary material, which is available to authorized users.

## Background

As one of the earliest centers of domestication for chickens, China has the most abundant chicken genetic resources in the world, with 107 indigenous chicken breeds. These breeds play an essential role in the Chinese poultry industry due to the popularity of traditional cuisine. Because these chickens typically exhibit high adaptability to variable environments, strong disease-resistance, and produce high-quality meat and eggs, they are an important breeding resource to meet future market demands [1]. However, a sizeable fraction of indigenous chicken breeds (21.3% in the world) are under threat of extinction because breeding populations are too small for genetic sustainability [2]. Twenty-three Chinese indigenous chicken breeds have been listed in the national conservation catalogue and are currently managed under what is thought to be an optimal conservation scheme. However, the effectiveness of the scheme has never been evaluated over the long term.

An efficient in situ conservation scheme relies on an effective population size, as well as an effective selection and mating strategy [1]. The recommended effective population size is 50, which is not only sufficient to maintain population fitness, but is also small enough to be monitored and managed easily [1, 3]. The principle of a selection and mating strategy is to minimize the average kinship between selected parents [1]. The mating systems that are in use are: (i) random mating and random selection (R: R), (ii) random mating within families, with one son kept per sire family and one daughter kept per dam family (R: F), and (iii) family rotational mating (F: R) [4].

Results from a study that compared the effectiveness of these three mating systems suggest that F: R can sustain 90% of genetic diversity in a livestock population for more than 100 years [4]. Other studies demonstrate that F: R can reduce inbreeding in populations [5, 6], however, implementation of the F: R scheme requires substantial effort. The contemporary conservation scheme in China relies mainly on the R: F mating system. Although simulation experiments suggest that R: F and F: R perform similarly in maintaining genetic diversity in a conserved population [4], few empirical studies have evaluated their effectiveness.

The genetic diversity dynamics in successive generations within a conserved population directly reflect the effectiveness of a conservation scheme. DNA markers can be used efficiently to estimate the genetic diversity within and between conserved populations [7]. Various DNA marker systems have been used to assess chicken genetic diversity, such as RAPDs [8], AFLPs [9], and microsatellites [10–12]. SNPs, which are densely distributed across genomes, have also been used to estimate genetic diversity and population structure with high accuracy [13, 14].

In order to investigate the effectiveness of the schemes that are currently used to conserve chicken genetic resources in China, we evaluated the genetic diversity of three conserved indigenous chicken breeds. SNPs, identified using high-throughput DNA sequencing in samples obtained from three generations per breed, were used to estimate diversity and track changes across time. These data can be used to evaluate and improve ongoing conservation efforts.

## Methods

### Ethics statement

Sample collection procedures strictly followed protocols approved by the Animal Welfare Committee of China Agricultural University (Approval Number: XK257).

### Sampling

Three Chinese indigenous chicken breeds that have been enrolled in conservation programs were used in this study: Baier Yellow Chicken (BEC), Beijing You Chicken (BYC), and Langshan Chicken (LSC). Different geographical and environmental factors have contributed to the unique characteristics of these breeds. The Baier Yellow chicken, mainly produced in Jiangxi Province, has a distinct appearance with white ears and yellow feathers, beak, and shanks. It is a rare prematurity and egg-type breed. The Langshan chicken is a classic dual-purpose breed that originated in Jiangsu Province. This is one of the oldest breeds and is unusually tall, with long legs and a tail carried at a high angle. The Beijing You chicken is an ancient breed that originated during the Qing Dynasty in Beijing. Known as “royal chickens”, they are valued for their high-quality meat and eggs, and are uniquely marked by a crest on the head, a beard under the lower jaw, and feathers on both shanks [15–17].

These breeds have been managed for optimal conservation as part of the National Chicken Genetics Resources program (Jiangsu, NCGR). Briefly, the conservation goals are that population sizes should be kept constant across generations (30 males and 300 females), and random mating should be enforced within families, with one son kept per sire family and one daughter kept per dam family (R: F). Samples from 270 individuals were collected from NCGR, with three generations per breed and 30 individuals per generation. Samples are identified by breed abbreviation and the last two digits of the year in which samples were obtained (e.g., samples collected from BEC in 2007 are designated BEC07). Breed and sampling information is summarized in Table 1. Blood samples were collected from the wing vein and stored at − 20 °C. Genomic DNA was extracted following the protocol accompanying the DNeasy Blood & Tissue Kit (Qiagen Inc., Valencia, California, USA). 3 μg high quality DNA was used to construct sequencing libraries for each sample.

**Table 1:** Samples obtained from conserved populations of three indigenous chicken breeds

Breed	Abbr.	Conservation initial year	Population size		Geographic location	Production type	Samples (*n*)		Years
			Sire	Dam			Sire	Dam	
Baier Yellow Chicken	BEC	1998	30	300	Jiangxi	Egg type	10	20	2007
			30	300			10	20	2010
			30	300			10	20	2015
Beijing You Chicken	BYC	1976	30	300	Beijing	Dual purpose	10	20	2007
			30	300			10	20	2010
			30	300			10	20	2015
Langshan Chicken	LSC	1998	30	300	Jiangsu	Dual purpose	10	20	2010
			30	300			10	20	2012
			30	300			10	20	2015

### Genotyping and data preparation

All DNA samples were subjected to genotyping by sequencing (GBS) using an Illumina HiSeq 4000 sequencer (Illumina, San Diego, CA, USA) after double enzyme digestion (*MseI* and *HaeIII*). The initial data set was filtered to exclude low-quality reads, and then aligned to the chicken genome (version: *Gallus_gallus 4.0*) using BWA (v0.7.8) [18]. PCR duplicates were removed using SAMtools rmdup (v0.1.19) [19]. Sequencing variants were identified using SAMtools mpileup (v0.1.19, arguments: -q 1 -C 50 -S -D -m 2 -F 0.002) and BCFtools view (arguments: -Q 20 -d 1 -D 8000). Variants satisfying all of the following criteria were retained for further analysis: coverage depth ≥ 1 and ≤ 8000, RMS mapping quality > 20, and distance between adjacent SNPs ≥5 bp. Variants were annotated using ANNOVAR with default parameters [20].

Quality control procedures were implemented using PLINK 1.90 [21]. SNPs were required to meet the following criteria: call rate ≥ 95%, minor allele frequency (MAF) ≥ 0.05, missing rate ≤ 0.01, and Hardy-Weinberg equilibrium test *P*-value >10e-6.

### Preparation of data prior to calculation of genetic diversity

Before genetic diversity was estimated, linkage disequilibrium (LD) “pruning” was conducted using PLINK (v1.90, arguments: --indep-pairwise 50 5 0.2).

Nine generation-based sample pools (BEC07, BEC10, BEC15; BYC07, BYC10, BEC13; LSC10, LSC12, LSC15) were used to calculate genetic diversity, as reflected by expected heterozygosity (*He*), observed heterozygosity (*Ho*), proportion of polymorphic markers (*P*_*N*_), and allelic richness (*A*_*R*_). *He*, *Ho* and *P*_*N*_ were calculated using PLINK 1.90 with the default settings. *A*_*R*_ estimates were determined using ADZE v1.0 [22].

### Inbreeding coefficient

Two measures of inbreeding coefficient were calculated for each chicken population.

Inbreeding coefficient based on the mating plan (*F*_ES_): The estimation of effective population size (*Ne*) was based on number of sires and dams, following Wright’s model [23]. Computation of *Ne* requires the numbers of males (*N*_m_) and females (N_f_) in each population that participated in the R: F program, and is calculated using the equation: $Ne=\frac{3 Nf+ Nm}{16\mathrm{NmNf}}$. The increment of hypothetical inbreeding (Δ*F*) was calculated using the equation: $\Delta F=\frac{1}{2 Ne}$.

Inbreeding coefficient based on runs of homozygosity (*F*_ROH_): A run of homozygosity is defined as a region > 100 Kb containing > 50 SNPs. *F*_ROH_ was calculated using PLINK v1.90 (with parameters --file BEC07_qc --ibc --allow-extra-chr --chr-set 28 --out BEC07) and is the fraction of the genome spanned by runs of homozygosity [24].

### Analysis of population structure

To reduce noise due to linkage disequilibrium, SNPs with a pair-wise genotype r^2^ value ≥0.2 were removed from the data set. A principal component analysis (PCA) [25] was conducted using PLINK and visualized with the SNPRelate R package [26]. A neighbor-joining (NJ) tree was constructed with Nei’s genetic distances [27] using the phylogeny program MEGA v7.0 [28] and displayed with FigTree v1.4.3 [29]. The genetic structures of the 9 sub-populations described above were analyzed with STRUCTURE v2.3.4 [30], using admixture and a correlated allele model [30, 31]. Ten independent runs were performed with K ranging from 1 to 10, with a burn-in period length of 10,000, followed by 100,000 Markov chain Monte Carlo (MCMC) repetitions, and 20 replications for each K value. STRUCTURE HARVESTER [32] was utilized to determine the optimal K value by comparing the likelihood of the data (LnK) for different values of K [lnP(X|K)] and by examining the second-order rate change of lnP(X|K),ΔK [33, 34]. Results for K = 2 to K = 9 are included in this report.

### Estimation of genetic differentiation

The unbiased genetic differentiation estimate, F_ST_ [35], was calculated using VCFtools v0.1.14 [36] with the quality-controlled SNP dataset to estimate genetic differentiation between populations (with parameters --vcf chicken_qc.vcf --weir-fst-pop BEC.txt --weir-fst-pop NLS.txt --out BEC_NLS).

### Estimation of nucleotide diversity

Genome-wide nucleotide diversity (π) was computed for each breed using VCFtools v0.1.14 [36] (parameters --vcf BEC_qc.recode.vcf --window-pi 100,000 --window-pi-step 10,000 --out BEC).

### Linkage disequilibrium decay

LD was evaluated as the correlation coefficient (r^2^) between alleles at two separate SNP loci [37]. Within each population, all pairs of autosomal SNPs with MAF > 0.05 and Hardy-Weinberg equilibrium *P-*value >10E-6 were used to calculate r^2^ with Haploview [38]. Inter-SNP distances from 0 kb to 500 kb were consolidated into 5 bins.

### Effective population size

Effective population size (*Ne*) was estimated according to the random mating model of linkage disequilibrium, using default parameters in N_E_ESTIMATOR v2.01 [39]. *Ne* estimates for each breed were calculated as the average of the estimates for macrochromosomes (gga1-gga5) [40] (Axelsson et al., 2005).

### Runs of homozygosity

Runs of homozygosity (ROH) were identified for each of the 9 sub-populations using PLINK v1.90. The ROH program slides a moving window of 1 Mb across the genome to estimate homozygosity. One heterozygous and five missing calls per window were allowed to avoid false negatives caused by occasional genotyping errors or missing genotypes. The minimum length and SNP counts required for each ROH were 100 kb and 50 SNPs, respectively. Additional statistical significant tests were conducted to detect the differences in genome-wide homozygosity levels among populations with three measures (NSEG, KB, KBAVG).

## Results

### Descriptive statistics

To assess genetic diversity, DNA samples from three indigenous chicken breeds (BEC, BYC, and LSC) were subjected to high-throughput DNA sequencing. Two hundred seventy individuals, representing three generations per breed and 30 individuals per generation, yielded 120 Gb of high-quality sequence data. About 99.7% of the reads mapped to the reference genome (*Gallus_gallus 4.0*) for each individual, providing ~8X average genome coverage. 6,950,965 SNPs were identified in the initial screen. 6,234,592 SNPs were excluded because they deviated from Hardy-Weinberg equilibrium (1,244,248 SNPs), exhibited minor allele frequency ≤ 0.05 (4,959,232 SNPs), or were located on non-autosomal or small chromosomes (31,112 SNPs). 716,373 SNPs met criteria for inclusion in the final data set. The average physical distance between neighboring SNPs was 1.34 kb, ranging from 1.10 kb on GGA6 to 5.15 kb on GGA25 (Additional file 1: Table S1). The distribution of SNPs across all chromosomes is shown in Additional file 2: Figure S1.

### Genetic diversity within the BEC, BYC and LSC breeds

**Fig. 1:**
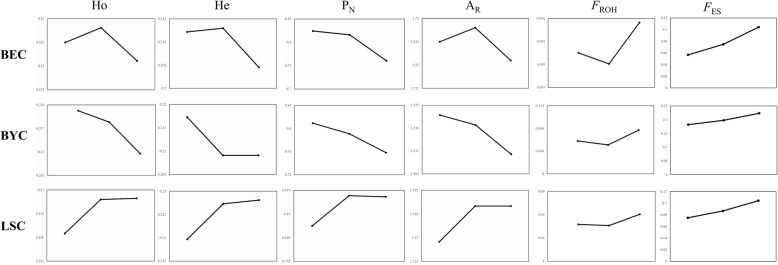
Dynamic changes between different generations within breeds. *Ho*, observed heterozygosity; *He*, expected heterozygosity; *P*_*N*_, proportion of polymorphic markers; *A*_*R*_, allelic richness; *F*_ROH,_ inbreeding coefficients based on ROH segments; *F*_ES_, inbreeding coefficient based on pedigree

All three breeds maintained relatively high genetic diversity in the R: F conservation scheme (see scheme definitions in Materials and Methods). LSC exhibited the highest genetic diversity as measured by *Ho* (0.2348), *He* (0.2250), *A*_*R*_ (1.235), and *P*_*N*_ (0.8130) (Table 2). An analysis across three generations within the same breed showed that genetic diversity was highest for LSC15 with *Ho* (0.2379)*, He* (0.2281) and *A*_*R*_ (1.238). The highest proportion of polymorphic markers (*P*_*N*_) was observed in BEC07 (82.41%), while BYC15 exhibited the lowest genetic diversity. As expected, BEC and BYC showed decreasing levels of diversity from the initial generation (BEC07/BYC07) to the current generation (BEC15/BYC15). Conversely, LSC displayed increasing diversity with the implementation of a conservation program (Fig. 1). However, dynamic changes in genetic diversity within breeds were less than 10% throughout the sampled generations (Additional file 3: Figure S2).

**Table 2:** Genetic diversity measurements for nine sub-populations from three chicken breeds

Population	Ho	He	P_N_ (%)	A_R_	*F*_ES_^a^	*F*_ROH_^b^
BEC07	0.2250	0.2195	82.41	1.225	0.0567	0.0500
BEC10	0.2281	0.2208	81.64	1.228	0.0748	0.0481
BEC15	0.2211	0.2073	76.06	1.221	0.1043	0.0553
BEC average	0.2247	0.2159	80.04	1.225	0.0789	0.0511
BYC07	0.2223	0.2173	80.95	1.222	0.1820	0.0698
BYC10	0.2189	0.2091	79.11	1.219	0.1978	0.0613
BYC15	0.2095	0.2091	75.83	1.210	0.2233	0.0925
BYC average	0.2169	0.2118	78.63	1.217	0.2010	0.0745
LSC10	0.2290	0.2197	80.55	1.229	0.0748	0.0474
LSC12	0.2376	0.2273	81.70	1.238	0.0867	0.0461
LSC15	0.2379	0.2281	81.66	1.238	0.1043	0.0606
LSC average	0.2348	0.2250	81.30	1.235	0.0886	0.0514

*He* Expected heterozygosity, *Ho* Observed heterozygosity, *P*_*N*_ Proportion of polymorphic SNPs, *A*_*R*_ Allelic richness, *F*_ES_^a^, inbreeding coefficient based on pedigree; *F*_ROH_^b^, inbreeding coefficient based on runs of homozygosity

### Estimation of inbreeding coefficients

Estimated inbreeding coefficients varied between breeds and conservation methods. Average *F*_ES_ ranged from 0.0789 in BEC to 0.2010 in BYC. As expected, *F*_ES_ values across generations increased as conservation procedures were maintained. In contrast, average *F*_ROH_ tended to be lower than *F*_ES_, ranging from 0.0511 in BEC to 0.0745 in BYC, and *F*_ROH_ did not exhibit the steady increase that was observed for *F*_ES_. Maximum inbreeding was observed in BYC15 (*F*_ES_ = 0.2010 and *F*_ROH_ = 0.0925). Correlation between *F*_ES_ and *F*_ROH_ was strongly positive (r^2^ = 0.76).

### Population structure analysis

**Fig. 2:**
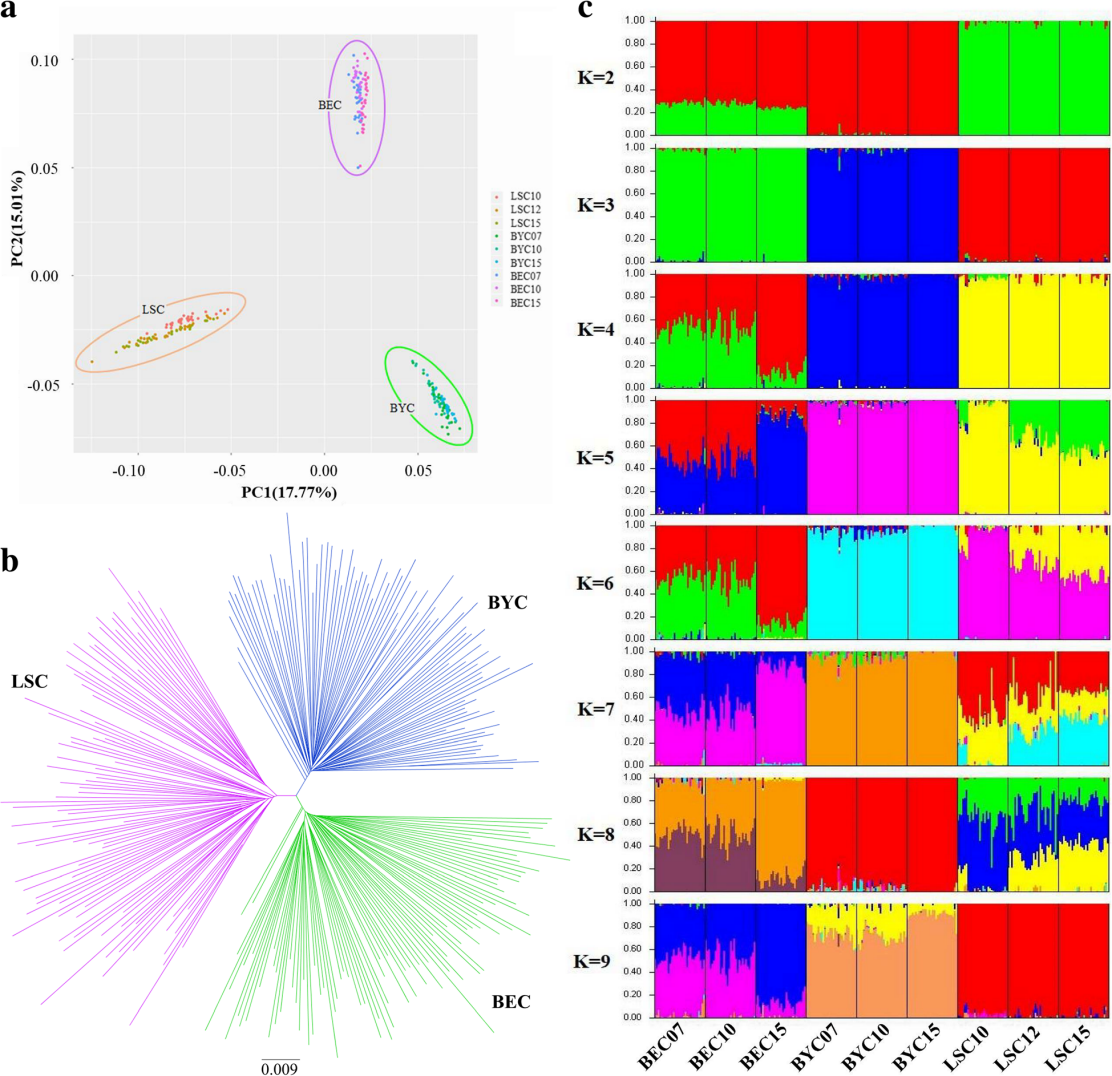
**a** Population structures of conserved populations revealed by principal component analysis. **b** Neighbor-joining tree constructed using genetic sharing distances. **c** Admixture plot for breeds analyzed based on different number of assumed ancestors (K). BEC, Baier Yellow Chicken; BYC, Beijing You Chicken; LSC, Langshan Chicken

Population structures of the three native chicken breeds, comprising 9 conservation sub-populations, were analyzed using PCA, NJ tree, and STRUCTURE. PCA showed that the first two principal components account for 17.77% (PC1) and 15.01% (PC2) of the total variability. Individuals from the 9 sub-populations clearly group into their respective breeds (Fig. 2a). The results of the NJ tree analysis were consistent with those obtained by PCA (Fig. 2b).

**Fig. 3:**
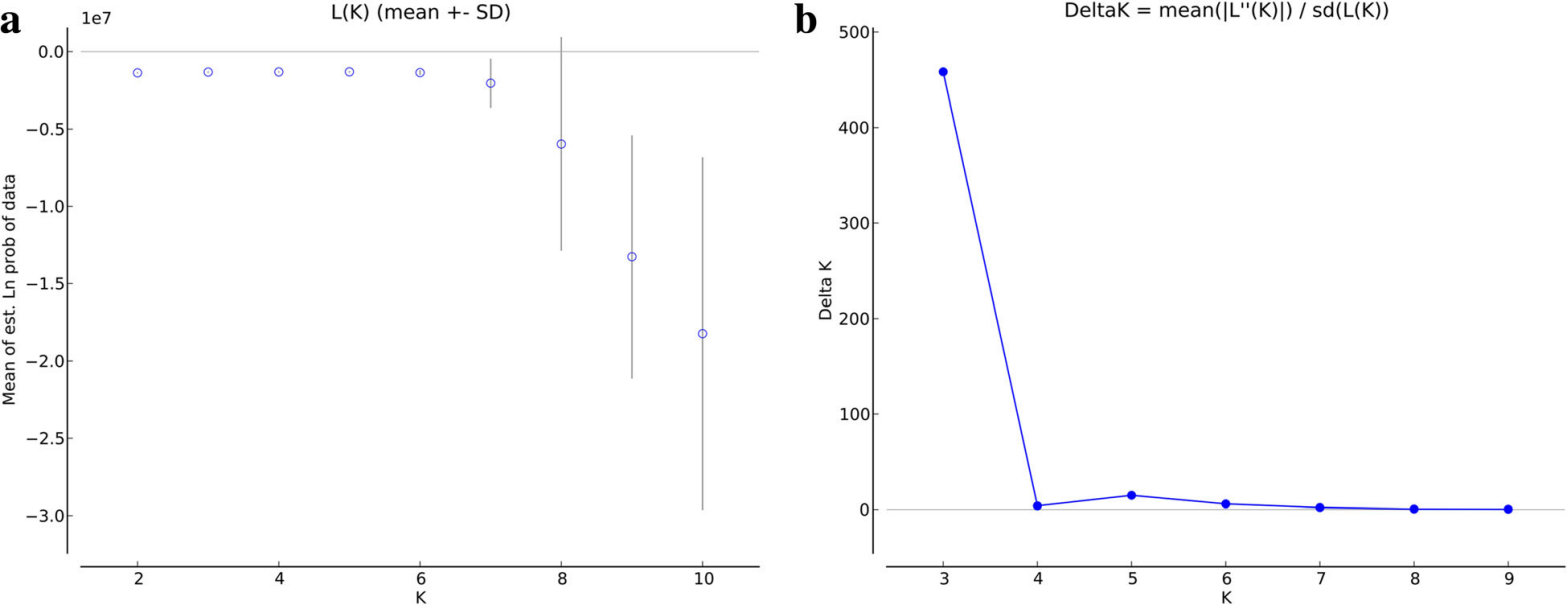
L(K) and ΔK values using different values of K, as calculated by STRUCTURE Harvester. **a** Average likelihood of runs in STRUCTURE L(K) along with number of K clusters. . **b** ΔK, estimator of the optimal number of clusters (K)

Figure 2c shows an admixture plot representing the 270 sampled chickens, generated using a model-based clustering approach. At a low value of K (K = 2), two distinct ancestors are apparent (BYC and LSC). BEC appears to include both LSC (as the majority component) and BYC. At K = 3, individuals cluster strongly into the three corresponding breeds, consistent with the PCA and NJ tree results (as shown in Fig. 2a and b). All generations within each breed show the same pattern. The optimum population structure inferred using the admixture model in STRUCTURE was subdivided into three sub-populations based on both LnP(D) and Evanno’s ∆K method (K = 3; Fig. 3). At K = 4, BEC splits into its two main ancestors. For K = 5 to 8, LSC appears to include two or more distinct ancestors, but at K = 9, it groups again into one common ancestor. Finally, the, BYC breed always exhibits homogeneity, except for K = 9.

### Population differentiation analysis

**Fig. 4:**
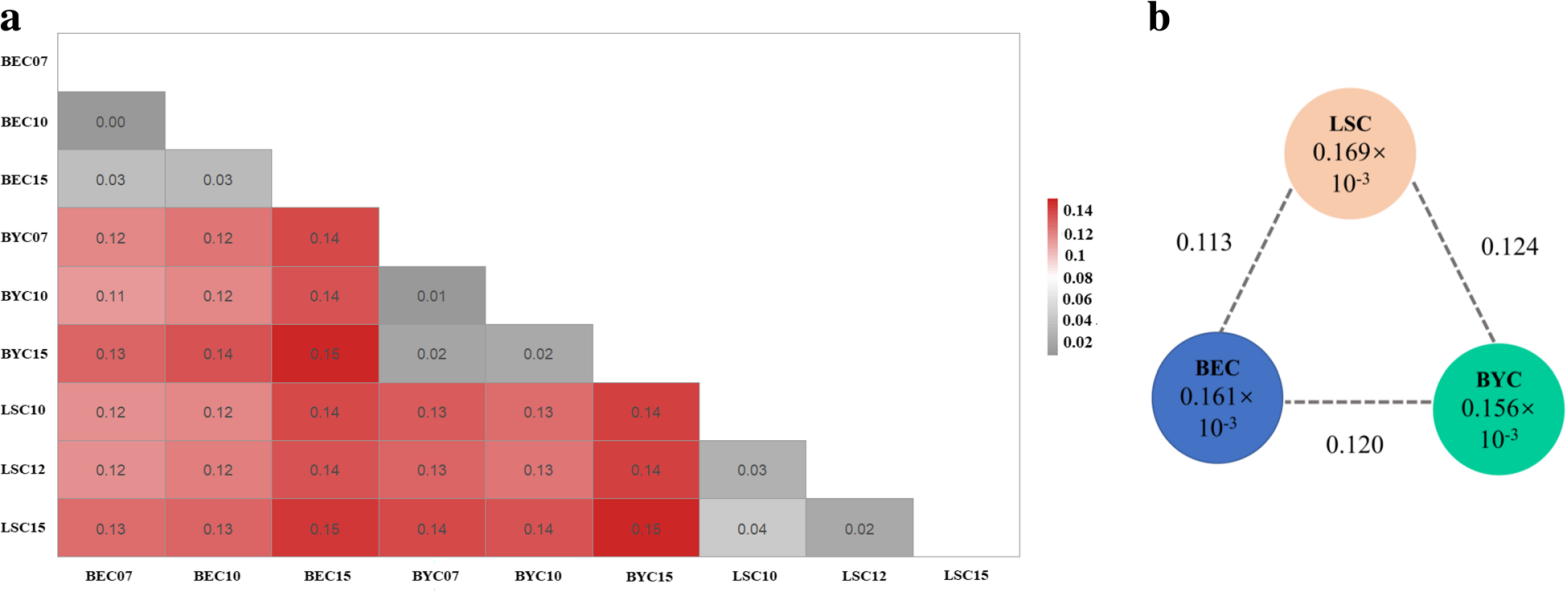
**a** Matrix showing pairwise differentiation estimates (*F*_ST_) between nine breed sub-populations. **b** Nucleotide diversity (π) and genetic differentiation (*F*_ST_) across the three breeds. The value in each circle represents a measure of nucleotide diversity for this breed, and the value on each line indicates genetic differentiation between the two breeds

To investigate the extent of population differentiation between different generations within breeds and between breeds, F_ST_ values were calculated using the filtered genotype data (Additional file 4: Table S2). The F_ST_ values for all pair-wise population comparisons are shown in Fig. 4a. For the entire population, F_ST_ values varied from 0.0046 to 0.1530, and F_ST_ values between breeds ranged from 0.1127 to 0.1243 (Fig. 4b). F_ST_ values are expected to be significantly higher between breeds than between generations within a breed. All F_ST_ values between generations within a breed were below 0.05 (from 0.0046 to 0.0423), indicating that no obvious genetic differentiation appeared within any breed (Fig. 4a). However, these F_ST_ values increased during conservation. For example, for BEC, the F_ST_ value was 0.0046 between BEC07 and BEC10, and increased to 0.0329 between BEC07 and BEC15. Similar trends were observed between BEC07 vs BEC15 (0.0329) and BEC10 vs BEC15 (0.0285).

### Linkage disequilibrium decay (LD decay) and effective population size

**Fig. 5:**
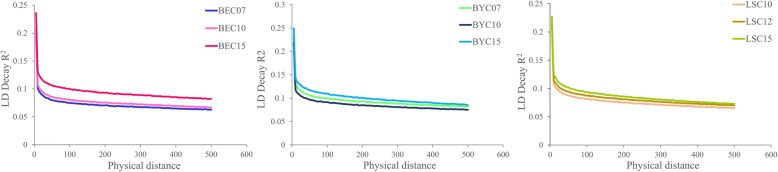
Linkage disequilibrium between generations and within each breed as a function of inter-SNP distance. Physical distance is measured in kb

LD for each population was estimated as the physical genomic distance at which the genotypic association (r^2^) decays to less than half of its maximum value. Short-range LD was always observed in each of the three different generations within same breed (Fig. 5). As expected, LD values tended to increase as conservation continued. For example, LD values for LSC10, 12 and 15 were 13.19 kb, 16.99 kb, and 20.10 kb, respectively. The BEC pattern was similar: 11.56 kb (BEC07), 13.55 kb (BEC10), and 20.77 kb (BEC15). The highest LD value in BYC occurred in the last generation (BYC15, 24.61 kb), but BYC07 (23.99 kb) exceeded BYC10 (17.95 kb).

Effective population size (*Ne*) was estimated for autosomal chromosomes gga1 through gga28 based on linkage disequilibrium (Table 3). Average *Ne* differed amongst the 9 sub-populations (from 19.33 to 34.85). Within macrochromosomes (gga1–5), BEC07 had the highest estimated *Ne* (81.52) within a breed, and *Ne* declined in BEC as conservation continued. In contrast, *Ne* was lower (55.36) in LSC12 than in LSC10 (70.28) or LSC15 (73.74). *Ne* estimates for BYC fluctuated, with a high value (75.04) in BYC10 and lower values in BYC07 and BYC15.

**Table 3:** Effective population sizes (*Ne*) for three Chinese indigenous chicken populations

Chromosome number	BEC			BYC			LSC		
	2007	2010	2015	2007	2010	2015	2010	2012	2015
Chr1	108.2	78.3	56.3	55.7	101.7	63.8	86.9	68.4	102.3
Chr2	96.4	66.6	51.4	55.5	91.0	60.6	63.5	56.8	78.9
Chr3	75.6	69.1	44.4	52.6	80.9	58.7	74.7	50.7	78.6
Chr4	70.8	53.7	28.1	34.8	51.7	57.5	63.6	56.6	63.9
Chr5	56.6	43.3	27.6	32.9	49.9	35.8	62.7	44.3	45.0
Chr6	36.9	27.9	25.9	22.3	36.0	25.6	52.1	31.9	28.3
Chr7	35.0	30.1	19.3	21.9	41.3	35.7	31.1	27.2	42.2
Chr8	30.7	37.1	22.5	16.8	30.5	20.9	34.6	32.8	32.9
Chr9	37.4	28.9	13.3	23.8	28.2	22.2	27.5	21.3	28.2
Chr10	21.4	21.7	9.1	15.1	20.9	17.2	25.4	25.0	20.9
Chr11	25.6	21.1	17.9	17.4	24.1	16.6	20.7	22.2	21.9
Chr12	39.9	29.3	23.2	19.0	30.5	24.9	31.6	30.4	25.4
Chr13	19.5	19.7	16.6	16.5	23.1	14.8	22.1	23.5	21.7
Chr14	26.0	21.7	16.6	14.8	23.5	20.8	25.5	26.2	19.6
Chr15	25.3	24.2	15.2	11.4	12.5	8.7	21.5	29.6	23.7
Chr16	3.3	5.5	3.0	2.4	3.1	2.6	2.9	4.1	2.6
Chr17	29.7	18.7	12.6	15.1	22.6	14.8	21.8	21.3	22.4
Chr18	19.2	19.8	13.3	15.6	17.7	17.4	20.5	15.5	15.7
Chr19	33.3	25.7	13.1	15.9	20.4	18.4	26.2	18.7	19.0
Chr20	24.7	24.6	15.9	8.8	16.3	14.0	26.2	22.8	18.1
Chr21	27.9	24.1	11.1	14.5	16.6	13.0	18.4	18.0	25.6
Chr22	10.5	9.7	6.9	7.5	11.5	9.0	16.4	17.2	15.9
Chr23	28.3	18.1	14.3	16.2	22.7	17.4	22.3	22.0	21.9
Chr24	20.5	16.6	11.2	14.2	19.6	16.1	19.2	23.2	17.5
Chr25	13.2	10.0	9.2	5.8	8.7	8.9	18.1	14.7	14.5
Chr26	18.8	15.6	12.7	9.4	18.5	12.2	24.5	14.9	16.5
Chr27	21.7	22.1	17.0	15.1	19.6	9.8	18.6	21.3	20.0
Chr28	19.3	14.8	13.4	12.0	20.1	15.0	22.7	16.0	17.4
Average (Chr1–5)	81.52	62.2	41.56	46.3	75.04	55.28	70.28	55.36	73.74
Average (Chr6–10)	32.28	29.14	18.02	19.98	31.38	24.32	34.14	27.64	30.5
Average (Chr11–28)	22.59	18.96	13.51	12.87	18.39	14.13	21.07	20.09	18.86
Average (all)	34.85	28.50	19.33	20.11	30.83	23.30	32.19	27.74	30.74

### Runs of homozygosity (ROH)

Runs of homozygosity (ROH) were identified in the genomes of the 9 sub-populations from all three breeds (Table 4). A genome-wide survey for autozygosity was conducted to identify regions with signatures that reflect ancient or recent inbreeding effects. We estimated *F*_ROH_, and found that the maximum values occurred in the BYC breed. All three BYC generations exceeded 0.05 (Table 2). BYC15 had the highest level of inbreeding (0.0925), while the BEC and LSC breeds had similar and lower inbreeding levels (~ 0.05).

**Table 4:** Statistical summary for runs of homozygosity in sub-populations of three chicken breeds

		9 sub-populations								
		BEC07	BEC10	BEC15	BYC07	BYC10	BYC15	LSC10	LSC12	LSC15
NSEG	Mean	283.3	276.9	359.93	434.93	365.03	535.2	302.73	286.2	336.37
	SD	29.79	37.14	76.71	68.13	41.16	74.15	47.20	38.37	51.53
	Min	198	203	233	262	285	362	245	208	238
	Max	331	374	528	555	459	710	441	385	426
KB	Mean	52,140.53	50,711.57	66,627.02	81,603.17	67,418.29	101,698.6	55,047.78	52,951.72	60,852.49
	SD	5327.89	6584.63	13,848.81	12,991.6	6958.4	14,048.53	8217.47	6859.34	9254.44
	Min	37,171.4	39,317.8	41,702.3	54,620.9	53,692.6	68,533.3	44,327.9	37,776.7	44,822.8
	Max	60,167.8	66,008.9	95,603.7	106,793	81,961.5	132,443	76,486.6	69,474.1	81,567.4
KB_AVER_	Mean	184.18	183.38	185.28	187.74	185	190.09	182.13	185.25	181.11
	SD	5.69	6.96	6.34	6.41	7.22	5.27	8.16	6.86	6.02
	Min	170.96	168.3	173.63	176.13	171.58	180.77	168.16	173.05	171.62
	Max	193.67	199.3	195.99	208.48	196.58	199.42	203.06	200.85	191.57
NSNP	Mean	87.92	88.78	91.13	93.25	89.96	96.28	87.76	86.45	88.98
	SD	32.75	33.13	34.27	37.12	34.76	38.44	32.35	31.1	32.6
	Min	50	50	50	50	50	50	50	50	50
	Max	368	339	332	383	370	419	392	310	343
Density	Mean	2.28	2.23	2.24	2.2	2.24	2.18	2.25	2.31	2.21
	SD	1.87	1.76	1.91	1.67	1.82	1.75	1.7	1.76	1.65
	Min	0.53	0.55	0.57	0.55	0.54	0.58	0.59	0.63	0.55
	Max	27.84	27.84	28.3	23.57	35.56	33.83	22.77	19.37	35.71
PHOM	Mean	0.99	0.99	0.99	0.995	0.99	0.995	0.99	0.99	0.99
	SD	0.01	0.01	0.01	0.01	0.01	0.01	0.01	0.01	0.01

NSEG average number of segments for the individual declared homozygous

KB average of total number of kb contained within homozygous segments

KB_AVER_ average size of homozygous segments

NSNP average number of SNPs in run

Density max inverse density (kb/SNP)

*PHOM* Proportion of sites homozygous

*Min* Minimum

*Max* Maximum

*SD* Standard deviation

**Fig. 6:**
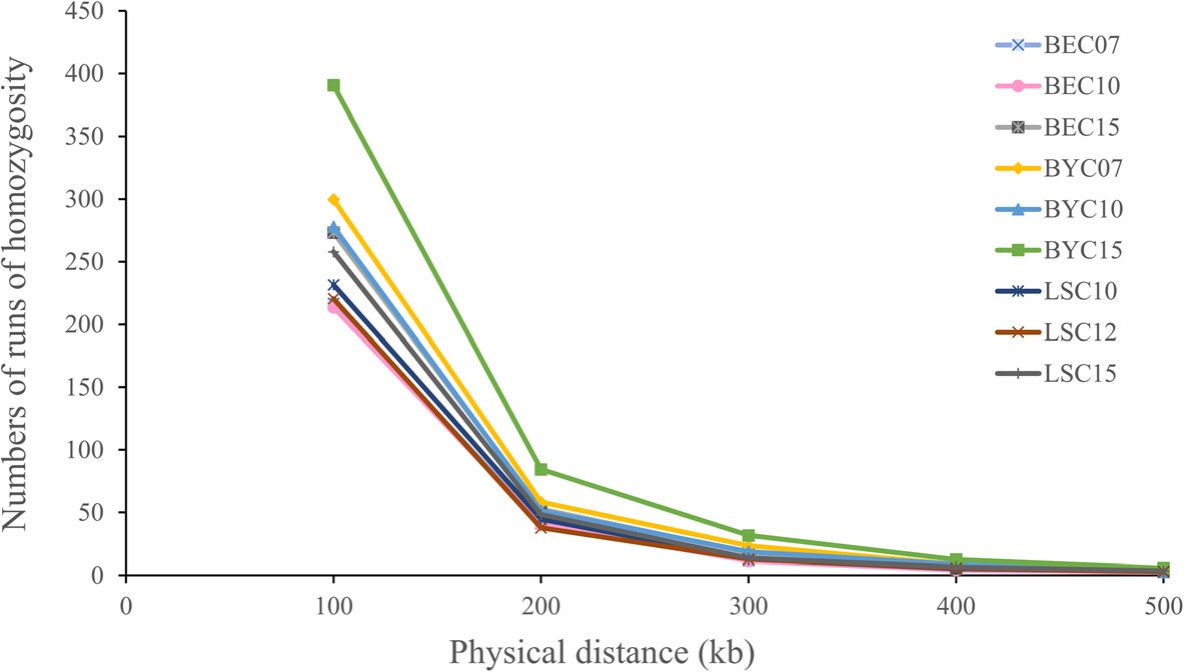
Homozygosity frequency distribution derived from runs of homozygosity (ROH) for each generation and breed

ROH was then assessed to determine whether any populations exhibited evidence of recent inbreeding. All three generations in BYC had higher ROH values, suggesting that recent inbreeding had occurred in this breed (Table 4 and Fig. 6). Consistent with the LD decay analysis, the highest ROH was observed in BYC15 (r^2^_0.1248_ = 24.61 kb), followed by BYC07 and BYC10. We speculate that BYC inbreeding might reflect the small effective population size of this breed (Table 3). The ROH values for BEC were highest in BEC15, followed by BEC07 and BEC10, which parallels the trend observed for LD decay and *F*_ROH_. Similar patterns were observed for LSC. Homozygosity was also measured between the individuals in each sub-population using three methods (NSEG, KB, and KB_AVER_) (Additional file 5: Table S3). All three measures varied significantly between BYC generations (*P*<0.01). NSEG and KB also showed significant differences (*P*<0.0001) between generations in BEC and NLS.

## Discussion

The chicken, one of the first animals to be domesticated, has been subjected to long-term natural selection, artificial selection, and genetic drift for diverse specific traits [41, 42]. A variety of factors accelerated the generation of phenotypic differences and genetic variability [43, 44]. However, this variability has been threatened due to ecosystem damage and commercial breeding. In China, gene pool (live) conservation and protected regions for both ex situ and in situ conservation have been established for the management of poultry genetic resources, as exemplified by the National Chicken Genetics Resources Program (Jiangsu). Because live conservation is typically implemented using small populations, it is necessary to monitor the status of each population to evaluate the effectiveness of the management strategy. In this study, we performed genotyping by sequencing (GBS) to assess the genomic diversity of different generations from three conserved breeds (Baier Yellow Chicken, Beijing You Chicken and Langshan Chicken).

The majority of studies have estimated genetic variability in Chinese indigenous chickens using microsatellites [45, 46] or mtDNA [47, 48], but genome-wide SNPs have seldomly been used. We estimated the genetic diversity in three chicken populations using SNP markers. The results showed that all three chicken breeds have maintained rich genetic diversity in terms of heterozygosity (*Ho*, *He*), proportion of polymorphic markers (*P*_*N*_), and allelic richness (*A*_*R*_), consistent with previous studies [45, 46, 49–51]. In the most recent generations sampled, LSC15 ranked first in genetic diversity, followed by BEC15 and BYC15 (Table 2). A study of the same populations in 2008 indicated that genetic diversity measured using microsatellites was highest in BYC, followed by LSC and BEC [45], suggesting that genetic diversity in BYC has decreased more rapidly than in the other breeds. We also observed this declining trend in the BYC breed. BYC diversity decreased from 2007 to 2015 (Table 2), while genetic variability in the BEC breed fluctuated, and LSC exhibited a slight increase. The BYC breed has been under conservation (~ 39 generations) for a longer period than either LSC or BEC, which have been conserved only since 1998 (~ 17 generations). The long-term practice of conservation in a small population size may reduce genetic diversity. Furthermore, all three breeds were subjected to ex situ live conservation in Jiangsu. The BYC breed, which originated in Beijing, might have adapted poorly to the environment, resulting in a loss of genetic diversity. In contrast, the LSC and BEC breeds might have adapted more easily.

The genetic diversity in all breeds changed no more than 10% between generations (Additional file 3: Figure S2). The conservation goal is to maintain 90% of the genetic diversity from the initial population and an inbreeding coefficient less than 0.1 for 100 years [52, 53]. According to our results, the genetic diversity of the three chicken populations meets conservation criteria under the current program (R: F). In particular, inbreeding events have been effectively avoided under the R: F mating system, based on assessments of population structure, genetic differentiation, LD decay, and ROH.

Nevertheless, the decline of genetic diversity should not be ignored (Fig. 1 and Table 2). The significant differences in ROH that we observe between generations in all three breeds also suggest that these populations have not reached the desired level of genetic stability during conservation. Both the decline in genetic diversity and the high heterozygosity across generations are indicative of genetic drift, which can be reduced by enlarging the population size. In our study, the estimated effective population sizes (*Ne*), based on whole-genome SNPs for the conserved populations, were far below the required threshold of 50 individuals [1, 3]. We also evaluated *Ne* according to chromosome size, using the classification proposed by the International Chicken Genome Sequencing Consortium [54]: large macrochromosomes (gga1–5), intermediate chromosomes (gga6–10) and micro-chromosomes (gga11–28). Because micro-chromosomes have high rates of recombination, we estimated *Ne* based on the macrochromosome class (gga1–5). The maximum *Ne* was 81.52 in BEC07 and the minimum was 41.56 in BEC15 (Table 3), suggesting these conserved populations are relatively stable but also at risk. We therefore recommend that ex situ live and in situ live conservation efforts be combined to help maintain high levels of genetic diversity in the long term.

## Conclusions

In summary, we collected 270 samples from three successive generations of three conserved chicken breeds. We estimated dynamic changes in genetic diversity using genome-wide SNPs, making it possible to comprehensively evaluate the current conservation scheme (R: F). The results demonstrated that the conserved Chinese chicken populations have sustained high levels of genetic variability under current conservation practices. We also compared successive generations within each breed to characterize trends in genetic diversity, allowing us to assess the effects of conservation over time. Overall, this study demonstrates an efficient strategy for assessing the success a conservation program and for improving conservation and management practices.

## Additional files

Additional file 1: **Table S1.** The distribution of SNPs and average distances between neighboring SNPs. (DOCX 17 kb)

Additional file 2: **Figure S1.** SNP density and distribution across the genome (after quality control). (TIF 2605 kb)

Additional file 3: **Figure S2.** Fluctuations in genetic diversity among different generations within each breed. (TIF 2872 kb)

Additional file 4: **Table S2.** Genetic differentiation (*F*_*ST*_ values) among the 9 sub-populations in three chicken breeds. (DOCX 25 kb)

Additional file 5: **Table S3.** Differences in measures of homozygosity between individuals among 9 sub-populations in three chicken breeds. (DOCX 27 kb)
