# Supplementary material for: Genomic diversity dynamics in conserved chicken populations are revealed by genome-wide SNPs
Source: BMC Genomics. 2018 Aug 9;19:598. doi: 10.1186/s12864-018-4973-6 (PMC6085637; doi:10.1186/s12864-018-4973-6)
Supplement: Supplementary file 1 — Table S1. The distribution of SNPs and average distances between neighboring SNPs. (DOCX 17 kb) [file 12864_2018_4973_MOESM1_ESM.docx]

Table S1. The distribution of the SNPs and the average distances between neighbored SNPs

| Chr | Size(Mbp) | Before QC SNPs | Mean distance between SNP(kb) | After QC SNPs | Mean distance between QC-SNP(kb) |
| --- | --- | --- | --- | --- | --- |
| Chr1 | 196.2 | 1415512 | 0.14 | 149114 | 1.32 |
| Chr2 | 149.6 | 1103887 | 0.14 | 118170 | 1.27 |
| Chr3 | 111.3 | 827300 | 0.13 | 87340 | 1.27 |
| Chr4 | 91.28 | 704629 | 0.13 | 75169 | 1.21 |
| Chr5 | 59.83 | 428420 | 0.14 | 43478 | 1.38 |
| Chr6 | 35.47 | 289476 | 0.12 | 32132 | 1.10 |
| Chr7 | 36.95 | 278257 | 0.13 | 30152 | 1.23 |
| Chr8 | 29.96 | 197954 | 0.15 | 21372 | 1.40 |
| Chr9 | 24.09 | 175572 | 0.14 | 19014 | 1.27 |
| Chr10 | 20.44 | 143781 | 0.14 | 16032 | 1.27 |
| Chr11 | 20.22 | 129157 | 0.16 | 13387 | 1.51 |
| Chr12 | 19.95 | 145670 | 0.14 | 15111 | 1.32 |
| Chr13 | 18.41 | 116286 | 0.16 | 12680 | 1.45 |
| Chr14 | 15.60 | 92404 | 0.17 | 9882 | 1.58 |
| Chr15 | 12.76 | 70360 | 0.18 | 7476 | 1.71 |
| Chr16 | 0.6523 | 1829 | 0.36 | 231 | 2.82 |
| Chr17 | 10.96 | 49826 | 0.22 | 5283 | 2.07 |
| Chr18 | 11.05 | 57493 | 0.19 | 6337 | 1.74 |
| Chr19 | 9.98 | 53953 | 0.18 | 6316 | 1.58 |
| Chr20 | 14.11 | 80242 | 0.18 | 8730 | 1.62 |
| Chr21 | 6.86 | 37305 | 0.18 | 4309 | 1.59 |
| Chr22 | 4.73 | 16813 | 0.28 | 1556 | 3.04 |
| Chr23 | 5.79 | 25039 | 0.23 | 2656 | 2.18 |
| Chr24 | 6.28 | 29956 | 0.21 | 3374 | 1.86 |
| Chr25 | 2.91 | 5112 | 0.57 | 565 | 5.15 |
| Chr26 | 5.31 | 20009 | 0.27 | 2196 | 2.42 |
| Chr27 | 5.66 | 18395 | 0.31 | 1929 | 2.93 |
| Chr28 | 4.97 | 15944 | 0.31 | 1742 | 2.85 |
| Total | 931.32 | 6530581 | 0.14 | 695733 | 1.34 |
