# Supplementary material for: Genomic diversity dynamics in conserved chicken populations are revealed by genome-wide SNPs
Source: BMC Genomics. 2018 Aug 9;19:598. doi: 10.1186/s12864-018-4973-6 (PMC6085637; doi:10.1186/s12864-018-4973-6)
Supplement: Supplementary file 4 — Table S2. Genetic differentiation (FST values) among the 9 sub-populations in three chicken breeds. (DOCX 25 kb) [file 12864_2018_4973_MOESM4_ESM.docx]

Table S2 Genetic differentiation (F_ST_ values) among the 9 sub-populations in three chicken breeds.

|  | BEC07 | BEC10 | BEC15 | BYC07 | BYC10 | BYC15 | LSC10 | LSC12 | LSC15 |
| --- | --- | --- | --- | --- | --- | --- | --- | --- | --- |
| BEC07 | 0 |  |  |  |  |  |  |  |  |
| BEC10 | 0.0046 | 0 |  |  |  |  |  |  |  |
| BEC15 | 0.0329 | 0.0285 | 0 |  |  |  |  |  |  |
| BYC07 | 0.1199 | 0.1249 | 0.1389 | 0 |  |  |  |  |  |
| BYC10 | 0.1141 | 0.1187 | 0.1361 | 0.0072 | 0 |  |  |  |  |
| BYC15 | 0.1317 | 0.1366 | 0.1515 | 0.0160 | 0.0179 | 0 |  |  |  |
| LSC10 | 0.1150 | 0.1187 | 0.1404 | 0.1324 | 0.1263 | 0.1434 | 0 |  |  |
| LSC12 | 0.1171 | 0.1212 | 0.1361 | 0.1308 | 0.1262 | 0.1431 | 0.0253 | 0 |  |
| LSC15 | 0.1271 | 0.1312 | 0.1452 | 0.1405 | 0.1366 | 0.1530 | 0.0423 | 0.0178 | 0 |
