# Supplementary material for: Genomic diversity dynamics in conserved chicken populations are revealed by genome-wide SNPs
Source: BMC Genomics. 2018 Aug 9;19:598. doi: 10.1186/s12864-018-4973-6 (PMC6085637; doi:10.1186/s12864-018-4973-6)
Supplement: Supplementary file 5 — Table S3. Differences in measures of homozygosity between individuals among 9 sub-populations in three chicken breeds. (DOCX 27 kb) [file 12864_2018_4973_MOESM5_ESM.docx]

Table S3 Differences in homozygosity measures between individuals among 9 sub-population in three chicken breeds.

| Measure | BEC | | BYC | | NLS | |
| --- | --- | --- | --- | --- | --- | --- |
|  | groups | P-value | groups | P-value | groups | P-value |
| NSEG | 07_10_15 | 0.0001 | 07_10_15 | 0.0001 | 10_12_15 | 0.0001 |
|  | 07_10 | 0.636 | 07_10 | 0.0001 | 10_12 | 0.168 |
|  | 10_15 | 0.0001 | 10_15 | 0.0001 | 12_15 | 0.0001 |
|  | 15_07 | 0.0001 | 15_07 | 0.0001 | 15_10 | 0.006 |
| KB | 07_10_15 | 0.0001 | 07_10_15 | 0.0001 | 10_12_15 | 0.001 |
|  | 07_10 | 0.556 | 07_10 | 0.0001 | 10_12 | 0.323 |
|  | 10_15 | 0.0001 | 10_15 | 0.0001 | 12_15 | 0.0001 |
|  | 15_07 | 0.0001 | 15_07 | 0.0001 | 15_10 | 0.007 |
| KB_AVER_ | 07_10_15 | 0.514 | 07_10_15 | 0.01 | 10_12_15 | 0.067 |
|  | 07_10 | 0.628 | 07_10 | 0.098 | 10_12 | 0.091 |
|  | 10_15 | 0.252 | 10_15 | 0.003 | 12_15 | 0.026 |
|  | 15_07 | 0.506 | 15_07 | 0.156 | 15_10 | 0.577 |
